# *rac*-2-(2-Chloro-6-methyl­quinolin-3-yl)-2,3-dihydro­quinolin-4(1*H*)-one

**DOI:** 10.1107/S1600536812020831

**Published:** 2012-05-12

**Authors:** Abdelmalek Bouraiou, Sofiane Bouacida, Carboni Bertrand, Thierry Roisnel, Ali Belfaitah

**Affiliations:** a Laboratoire des Produits Naturels d’Origine Végétale et de Synthèse Organique (PHYSYNOR), Université Mentouri-Constantine, 25000 Constantine, Algeria; b Unité de Recherche de Chimie de l’Environnement et Moléculaire Structurale (CHEMS), Université Mentouri-Constantine, 25000 Algeria; c UMR 6226 CNRS Sciences Chimiques de Rennes, Université de Rennes I, France; d Centre de Difractométrie X, UMR 6226 CNRS Unité Sciences Chimiques de Rennes, Université de Rennes I, 263 Avenue du Général Leclerc, 35042 Rennes, France

## Abstract

In the title compound, C_19_H_15_ClN_2_O, the quinoline ring forms a dihedral angle of 43.24 (1)° with the benzene ring of the dihydroquinolinyl system. In the crystal, mol­ecules are linked through a single weak C—H⋯O hydrogen bond, forming ribbons which extend along (100), giving alternating zigzag mol­ecular layers which stack down the *b*-axis direction.

## Related literature

For applications of similar structures see: Chandrasekhar *et al.* (2007 ▶); Varma & Saini (1997 ▶); Donnelly & Farrell (1990 ▶); Hemanth Kumar *et al.* (2004 ▶). For the synthesis of the 2-amino­chalcone, see: Gao *et al.* (1996 ▶). For related structures, see: Bouraiou *et al.* (2008 ▶, 2011 ▶); Belfaitah *et al.* (2006 ▶); Benzerka *et al.* (2011 ▶).
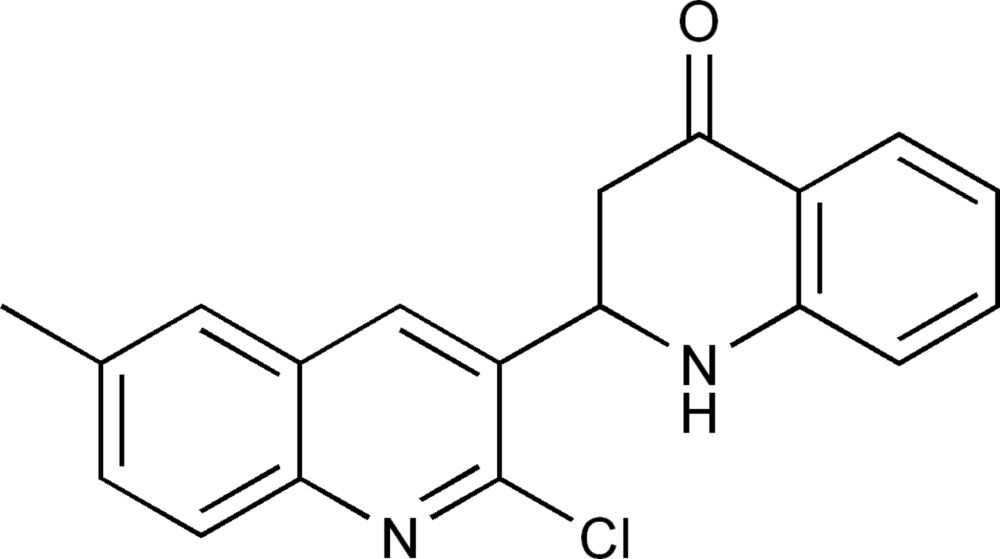


## Experimental

### 

#### Crystal data

C_19_H_15_ClN_2_O
*M* *_r_* = 322.78
Orthorhombic, 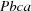

*a* = 13.8912 (8) Å
*b* = 12.4572 (4) Å
*c* = 17.8617 (11) Å
*V* = 3090.9 (3) Å^3^
*Z* = 8
Mo *K*α radiation
μ = 0.25 mm^−1^
*T* = 295 K
0.15 × 0.06 × 0.05 mm

#### Data collection

Nonius KappaCCD diffractometer
6664 measured reflections
3537 independent reflections
1696 reflections with *I* > 2σ(*I*)
*R*_int_ = 0.072

#### Refinement

*R*[*F* ^2^ > 2σ(*F* ^2^)] = 0.062
*wR*(*F* ^2^) = 0.169
*S* = 1.00
3537 reflections
212 parameters
H atoms treated by a mixture of independent and constrained refinement
Δρ_max_ = 0.21 e Å^−3^
Δρ_min_ = −0.27 e Å^−3^

### 

Data collection: *COLLECT* (Nonius, 1998 ▶); cell refinement: *SCALEPACK* (Otwinowski & Minor, 1997 ▶); data reduction: *DENZO* (Otwinowski & Minor, 1997 ▶) and *SCALEPACK*; program(s) used to solve structure: *SIR2002* (Burla *et al.*, 2003 ▶); program(s) used to refine structure: *SHELXL97* (Sheldrick, 2008 ▶); molecular graphics: *ORTEP-3* (Farrugia,1997 ▶) and *DIAMOND* (Brandenburg & Berndt, 2001 ▶); software used to prepare material for publication: *WinGX* (Farrugia, 1999 ▶).

## Supplementary Material

Crystal structure: contains datablock(s) global, I. DOI: 10.1107/S1600536812020831/zs2200sup1.cif

Structure factors: contains datablock(s) I. DOI: 10.1107/S1600536812020831/zs2200Isup2.hkl

Supplementary material file. DOI: 10.1107/S1600536812020831/zs2200Isup3.cml

Additional supplementary materials:  crystallographic information; 3D view; checkCIF report

## Figures and Tables

**Table 1:** Hydrogen-bond geometry (Å, °)

*D*—H⋯*A*	*D*—H	H⋯*A*	*D*⋯*A*	*D*—H⋯*A*
C17—H17⋯O1^i^	0.93	2.49	3.243 (5)	138

Symmetry code: (i) 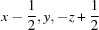.

**Fig. 1.**
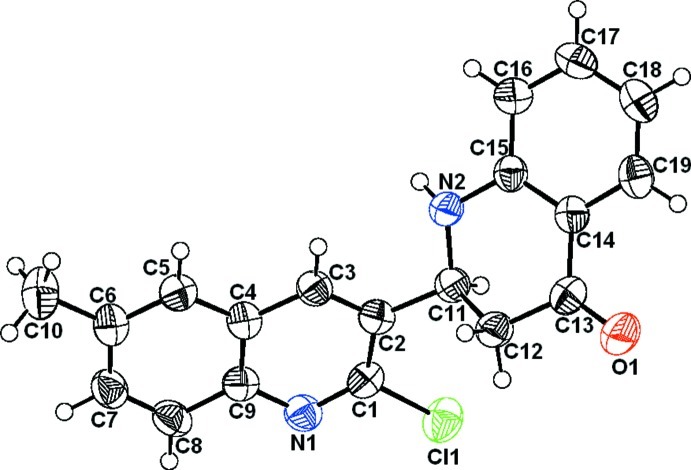
The structure of the title compound with the atom-labelling scheme. Displacement ellipsoids are drawn at the 50% probability level.

**Fig. 2.**
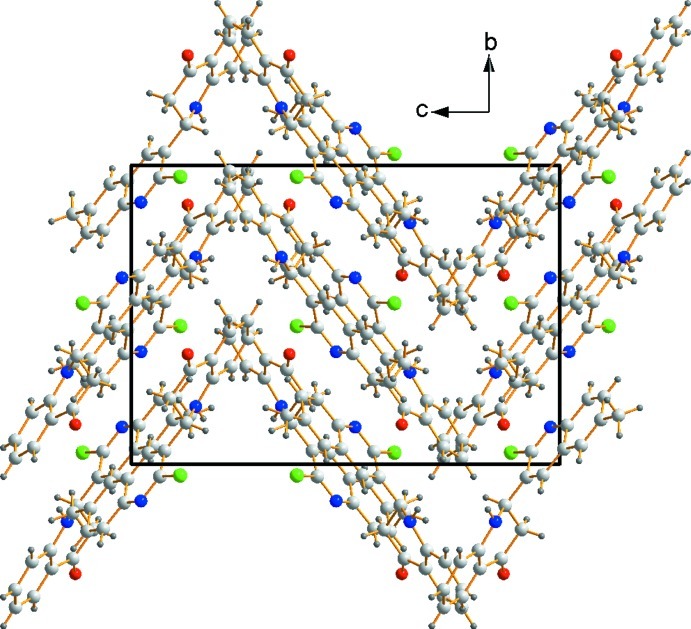
A diagram of the layered zigzag packing in the crystal viewed down the a axis.

**Fig. 3.**
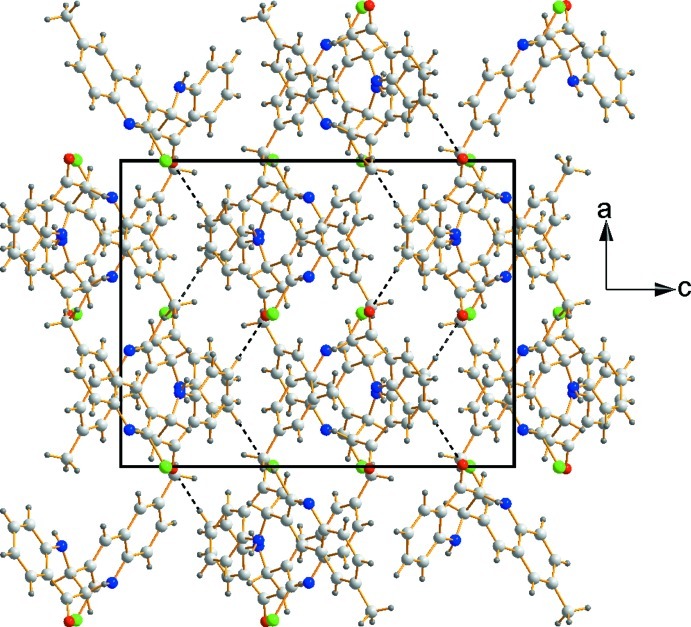
A part of crystal packing viewed down the b axis showing hydrogen-bond interactions as dashed lines.

## supplementary crystallographic information

### Comment

2-Substituted dihydroquinolinones have important medicinal properties as new chemical entities and also serve as building blocks for creating further diversity in SAR studies in various therapeutic areas (Chandrasekhar *et al.*, 2007). Convenient synthesis of 2-aminochalcone and its amide derivatives and the ready cyclization of these compounds to 2-aryl-2,3-dihydroquinolin-4(1*H*)-ones have been widely explored (Varma & Saini, 1997; Donnelly & Farrell, 1990). Silica gel supported InCl_3_ (20 mol %) is a new solid-support catalyst that can be used under solvent-free conditions for the facile and efficient isomerization of 2-aminochalcones to the corresponding 2-aryl-2,3-dihydroquinolin-4(1*H*)-ones (Hemanth Kumar *et al.*, 2004). As a part of a program directed toward the synthesis of new suitably functionalized heterocyclic compounds of potential biological activity (Bouraiou *et al.*, 2008, 2011; Benzerka *et al.*, 2011), we report herein the synthesis and structure determination of the title compound, C_19_H_15_ClN_2_ O.

In the title compound (Fig. 1), the quinoline ring forms a dihedral angle of 43.24 (1)° with the phenyl ring of the 2,3-dihydroquinolin-4(1*H*)-one moiety. The geometric parameters are in agreement with those of other structures possessing a quinolyl substituent, previously reported in the literature (Belfaitah *et al.*, 2006; Benzerka *et al.*, 2011). The crystal structure can be described as alterning zigzag ribbons which stack down the *b* axis of the unit cell (Fig. 2), these ribbons comprising molecules linked through a single weak intermolecular C17—H···O1 hydrogen bond (Table 1), and extending down *a* (Fig. 3). The hetero N2—H2 group has no acceptor in the crystal structure.

### Experimental

2-Aminoacetophenone (1mmol) was first condensed with 2-chloro-3-formyl-6-methylquinoline (2 mmol) to give the corresponding 2-aminochalcone in 74% yield, according to the procedure described by Gao *et al.* (1996). In the next step, a mixture of 2-aminochalcone (100 mg) and 1 g of silica gel impregnated with indium(III) chloride (20 mol%, 13.6 mg) was irradiated in a domestic microwave oven at 360 W for 5 minutes. Under these conditions, the title compound was successfully synthesized in good yield (69%). Single crystals suitable for the X-ray diffraction analysis were obtained by dissolving the compound in a diisopropyl ether/CHCl_3_ solvent mixture and allowing the solution to slowly evaporate at room temperature.

### Refinement

The N-bound H-atom (H2) was located in a difference-Fourier map and its positional parameters were refined isotropically. All other H atoms were introduced in calculated positions and treated as riding on their parent C atom, with C—H = 0.93, 0.96, 0.97 or 0.98 Å, with *U*_iso_(H) = 1.2 or 1.5 *U*_eq_(C). No H-bond acceptor could be located for the N2—H2 group.

### Crystal data

**Table:**

C_19_H_15_ClN_2_O	*F*(000) = 1344
*M**_r_* = 322.78	*D*_x_ = 1.387 Mg m^−^^3^
Orthorhombic, *P**b**c**a*	Mo *K*α radiation, λ = 0.71073 Å
Hall symbol: -P 2ac 2ab	Cell parameters from 3949 reflections
*a* = 13.8912 (8) Å	θ = 2.9–27.5°
*b* = 12.4572 (4) Å	µ = 0.25 mm^−^^1^
*c* = 17.8617 (11) Å	*T* = 295 K
*V* = 3090.9 (3) Å^3^	Needle, colourless
*Z* = 8	0.15 × 0.06 × 0.05 mm

### Data collection

**Table:**

Nonius KappaCCD diffractometer	1696 reflections with *I* > 2σ(*I*)
Radiation source: Enraf Nonius FR590 diffractometer	*R*_int_ = 0.072
Graphite monochromator	θ_max_ = 27.5°, θ_min_ = 2.9°
Detector resolution: 9 pixels mm^-1^	*h* = −18→17
CCD rotation images, thick slices scans	*k* = −16→16
6664 measured reflections	*l* = −23→23
3537 independent reflections	

### Refinement

**Table:**

Refinement on *F*^2^	Primary atom site location: structure-invariant direct methods
Least-squares matrix: full	Secondary atom site location: difference Fourier map
*R*[*F*^2^ > 2σ(*F*^2^)] = 0.062	Hydrogen site location: inferred from neighbouring sites
*wR*(*F*^2^) = 0.169	H atoms treated by a mixture of independent and constrained refinement
*S* = 1.00	*w* = 1/[σ^2^(*F*_o_^2^) + (0.0676*P*)^2^ + 0.5198*P*] where *P* = (*F*_o_^2^ + 2*F*_c_^2^)/3
3537 reflections	(Δ/σ)_max_ < 0.001
212 parameters	Δρ_max_ = 0.21 e Å^−^^3^
0 restraints	Δρ_min_ = −0.27 e Å^−^^3^

### Special details

**Table:**

Geometry. All e.s.d.'s (except the e.s.d. in the dihedral angle between two l.s. planes) are estimated using the full covariance matrix. The cell e.s.d.'s are taken into account individually in the estimation of e.s.d.'s in distances, angles and torsion angles; correlations between e.s.d.'s in cell parameters are only used when they are defined by crystal symmetry. An approximate (isotropic) treatment of cell e.s.d.'s is used for estimating e.s.d.'s involving l.s. planes.
Refinement. Refinement of *F*^2^ against ALL reflections. The weighted *R*-factor *wR* and goodness of fit *S* are based on *F*^2^, conventional *R*-factors *R* are based on *F*, with *F* set to zero for negative *F*^2^. The threshold expression of *F*^2^ > σ(*F*^2^) is used only for calculating *R*-factors(gt) *etc*. and is not relevant to the choice of reflections for refinement. *R*-factors based on *F*^2^ are statistically about twice as large as those based on *F*, and *R*- factors based on ALL data will be even larger.

### Fractional atomic coordinates and isotropic or equivalent isotropic displacement parameters (Å^2^)

**Table:**

	*x*	*y*	*z*	*U*_iso_*/*U*_eq_	
Cl1	1.00123 (7)	0.53833 (7)	0.38618 (6)	0.0598 (3)	
O1	1.00620 (19)	0.1322 (2)	0.36882 (17)	0.0724 (9)	
N1	0.8795 (2)	0.6237 (2)	0.47797 (17)	0.0467 (7)	
N2	0.7597 (2)	0.3073 (2)	0.34987 (16)	0.0461 (7)	
C1	0.8911 (2)	0.5416 (2)	0.43385 (19)	0.0430 (8)	
C2	0.8250 (2)	0.4560 (2)	0.42234 (18)	0.0408 (7)	
C3	0.7405 (2)	0.4630 (2)	0.46099 (18)	0.0430 (8)	
H3	0.6944	0.4096	0.4554	0.052*	
C4	0.7221 (2)	0.5504 (2)	0.50953 (18)	0.0411 (8)	
C5	0.6356 (2)	0.5626 (2)	0.54971 (19)	0.0453 (8)	
H5	0.5879	0.5108	0.5447	0.054*	
C6	0.6196 (3)	0.6486 (2)	0.59604 (19)	0.0474 (9)	
C7	0.6938 (3)	0.7251 (3)	0.60359 (19)	0.0515 (9)	
H7	0.6842	0.7834	0.6353	0.062*	
C8	0.7791 (3)	0.7169 (2)	0.56608 (19)	0.0508 (9)	
H8	0.8269	0.7682	0.5728	0.061*	
C9	0.7940 (2)	0.6298 (2)	0.51708 (19)	0.0424 (8)	
C10	0.5261 (3)	0.6631 (3)	0.6368 (2)	0.0687 (12)	
H10A	0.4871	0.7146	0.6107	0.103*	
H10B	0.5385	0.6883	0.6867	0.103*	
H10C	0.4927	0.5957	0.6391	0.103*	
C11	0.8477 (2)	0.3617 (2)	0.37226 (19)	0.0442 (8)	
H11	0.8803	0.3883	0.3273	0.053*	
C12	0.9131 (3)	0.2810 (2)	0.41094 (19)	0.0479 (9)	
H12A	0.9741	0.3151	0.4226	0.058*	
H12B	0.8838	0.2588	0.4577	0.058*	
C13	0.9313 (3)	0.1834 (3)	0.3631 (2)	0.0475 (8)	
C14	0.8524 (2)	0.1510 (2)	0.31357 (18)	0.0420 (8)	
C15	0.7678 (2)	0.2110 (2)	0.31041 (18)	0.0412 (8)	
C16	0.6899 (3)	0.1735 (3)	0.2677 (2)	0.0523 (9)	
H16	0.633	0.2127	0.266	0.063*	
C17	0.6976 (3)	0.0793 (3)	0.2284 (2)	0.0534 (9)	
H17	0.6453	0.0546	0.2008	0.064*	
C18	0.7825 (3)	0.0204 (3)	0.2292 (2)	0.0559 (9)	
H18	0.7878	−0.0423	0.2012	0.067*	
C19	0.8584 (3)	0.0556 (2)	0.2717 (2)	0.0505 (9)	
H19	0.915	0.0157	0.2728	0.061*	
H2	0.717 (3)	0.348 (2)	0.3321 (19)	0.05*	

### Atomic displacement parameters (Å^2^)

**Table:**

	*U*^11^	*U*^22^	*U*^33^	*U*^12^	*U*^13^	*U*^23^
Cl1	0.0485 (5)	0.0545 (5)	0.0763 (7)	−0.0051 (4)	0.0144 (5)	0.0027 (5)
O1	0.0539 (18)	0.0643 (16)	0.099 (2)	0.0201 (13)	−0.0134 (16)	−0.0173 (15)
N1	0.0479 (18)	0.0388 (14)	0.0534 (17)	−0.0029 (13)	−0.0024 (14)	−0.0010 (13)
N2	0.0408 (17)	0.0419 (15)	0.0554 (18)	0.0075 (12)	−0.0080 (14)	−0.0077 (13)
C1	0.0382 (19)	0.0412 (17)	0.050 (2)	0.0007 (14)	0.0014 (15)	0.0067 (15)
C2	0.0450 (19)	0.0353 (16)	0.0421 (18)	0.0008 (14)	−0.0046 (16)	0.0041 (14)
C3	0.045 (2)	0.0360 (16)	0.0482 (19)	−0.0027 (14)	−0.0011 (16)	−0.0015 (15)
C4	0.047 (2)	0.0352 (15)	0.0412 (18)	0.0007 (15)	0.0025 (15)	0.0043 (14)
C5	0.047 (2)	0.0378 (16)	0.051 (2)	−0.0021 (14)	−0.0016 (17)	0.0033 (15)
C6	0.057 (2)	0.0407 (17)	0.045 (2)	0.0054 (16)	0.0057 (17)	0.0048 (15)
C7	0.062 (2)	0.0438 (18)	0.049 (2)	0.0108 (17)	−0.0084 (19)	−0.0058 (16)
C8	0.059 (2)	0.0394 (17)	0.053 (2)	−0.0029 (16)	−0.0091 (19)	−0.0063 (16)
C9	0.043 (2)	0.0382 (16)	0.0454 (19)	−0.0004 (14)	−0.0040 (16)	0.0028 (14)
C10	0.075 (3)	0.054 (2)	0.078 (3)	0.007 (2)	0.022 (2)	−0.004 (2)
C11	0.042 (2)	0.0401 (17)	0.050 (2)	0.0005 (14)	−0.0020 (16)	−0.0038 (15)
C12	0.047 (2)	0.0439 (18)	0.053 (2)	0.0052 (15)	−0.0083 (17)	−0.0013 (15)
C13	0.042 (2)	0.0461 (18)	0.054 (2)	0.0036 (16)	0.0013 (17)	0.0044 (16)
C14	0.044 (2)	0.0397 (16)	0.0423 (19)	−0.0002 (14)	0.0043 (15)	0.0014 (14)
C15	0.042 (2)	0.0396 (16)	0.0419 (18)	0.0000 (14)	0.0038 (15)	0.0004 (14)
C16	0.047 (2)	0.055 (2)	0.055 (2)	−0.0028 (16)	−0.0017 (17)	−0.0058 (17)
C17	0.060 (3)	0.053 (2)	0.047 (2)	−0.0127 (17)	−0.0048 (19)	−0.0062 (16)
C18	0.071 (3)	0.0454 (18)	0.051 (2)	−0.0023 (18)	0.005 (2)	−0.0066 (16)
C19	0.061 (2)	0.0423 (17)	0.048 (2)	0.0054 (16)	0.0067 (19)	0.0001 (16)

### Geometric parameters (Å, º)

**Table:**

Cl1—C1	1.751 (3)	C8—H8	0.93
O1—C13	1.224 (4)	C10—H10A	0.96
N1—C1	1.301 (4)	C10—H10B	0.96
N1—C9	1.380 (4)	C10—H10C	0.96
N2—C15	1.395 (4)	C11—C12	1.522 (4)
N2—C11	1.454 (4)	C11—H11	0.98
N2—H2	0.85 (3)	C12—C13	1.507 (4)
C1—C2	1.423 (4)	C12—H12A	0.97
C2—C3	1.364 (5)	C12—H12B	0.97
C2—C11	1.509 (4)	C13—C14	1.466 (5)
C3—C4	1.415 (4)	C14—C15	1.395 (4)
C3—H3	0.93	C14—C19	1.406 (4)
C4—C5	1.407 (5)	C15—C16	1.404 (4)
C4—C9	1.412 (4)	C16—C17	1.372 (4)
C5—C6	1.372 (4)	C16—H16	0.93
C5—H5	0.93	C17—C18	1.389 (5)
C6—C7	1.410 (5)	C17—H17	0.93
C6—C10	1.500 (5)	C18—C19	1.372 (5)
C7—C8	1.365 (5)	C18—H18	0.93
C7—H7	0.93	C19—H19	0.93
C8—C9	1.409 (4)		
			
C1—N1—C9	117.2 (3)	H10A—C10—H10C	109.5
C15—N2—C11	118.2 (3)	H10B—C10—H10C	109.5
C15—N2—H2	112 (2)	N2—C11—C2	110.5 (3)
C11—N2—H2	115 (2)	N2—C11—C12	108.6 (3)
N1—C1—C2	126.6 (3)	C2—C11—C12	111.7 (3)
N1—C1—Cl1	114.9 (2)	N2—C11—H11	108.7
C2—C1—Cl1	118.4 (2)	C2—C11—H11	108.7
C3—C2—C1	115.7 (3)	C12—C11—H11	108.7
C3—C2—C11	122.0 (3)	C13—C12—C11	112.1 (3)
C1—C2—C11	122.3 (3)	C13—C12—H12A	109.2
C2—C3—C4	121.1 (3)	C11—C12—H12A	109.2
C2—C3—H3	119.5	C13—C12—H12B	109.2
C4—C3—H3	119.5	C11—C12—H12B	109.2
C5—C4—C9	118.7 (3)	H12A—C12—H12B	107.9
C5—C4—C3	123.4 (3)	O1—C13—C14	122.8 (3)
C9—C4—C3	118.0 (3)	O1—C13—C12	121.0 (3)
C6—C5—C4	122.0 (3)	C14—C13—C12	116.1 (3)
C6—C5—H5	119	C15—C14—C19	118.8 (3)
C4—C5—H5	119	C15—C14—C13	120.5 (3)
C5—C6—C7	117.9 (3)	C19—C14—C13	120.6 (3)
C5—C6—C10	121.8 (3)	C14—C15—N2	120.5 (3)
C7—C6—C10	120.3 (3)	C14—C15—C16	119.5 (3)
C8—C7—C6	122.4 (3)	N2—C15—C16	119.9 (3)
C8—C7—H7	118.8	C17—C16—C15	120.2 (3)
C6—C7—H7	118.8	C17—C16—H16	119.9
C7—C8—C9	119.4 (3)	C15—C16—H16	119.9
C7—C8—H8	120.3	C16—C17—C18	120.9 (3)
C9—C8—H8	120.3	C16—C17—H17	119.6
N1—C9—C8	118.9 (3)	C18—C17—H17	119.6
N1—C9—C4	121.5 (3)	C19—C18—C17	119.3 (3)
C8—C9—C4	119.6 (3)	C19—C18—H18	120.4
C6—C10—H10A	109.5	C17—C18—H18	120.4
C6—C10—H10B	109.5	C18—C19—C14	121.3 (3)
H10A—C10—H10B	109.5	C18—C19—H19	119.4
C6—C10—H10C	109.5	C14—C19—H19	119.4
			
C9—N1—C1—C2	−1.0 (5)	C15—N2—C11—C12	−50.0 (4)
C9—N1—C1—Cl1	−179.8 (2)	C3—C2—C11—N2	21.5 (4)
N1—C1—C2—C3	1.2 (5)	C1—C2—C11—N2	−160.2 (3)
Cl1—C1—C2—C3	180.0 (2)	C3—C2—C11—C12	−99.5 (4)
N1—C1—C2—C11	−177.2 (3)	C1—C2—C11—C12	78.8 (4)
Cl1—C1—C2—C11	1.6 (4)	N2—C11—C12—C13	54.4 (4)
C1—C2—C3—C4	−0.5 (5)	C2—C11—C12—C13	176.5 (3)
C11—C2—C3—C4	177.9 (3)	C11—C12—C13—O1	151.2 (3)
C2—C3—C4—C5	178.9 (3)	C11—C12—C13—C14	−32.2 (4)
C2—C3—C4—C9	−0.4 (5)	O1—C13—C14—C15	178.7 (3)
C9—C4—C5—C6	−0.4 (5)	C12—C13—C14—C15	2.1 (5)
C3—C4—C5—C6	−179.6 (3)	O1—C13—C14—C19	2.3 (5)
C4—C5—C6—C7	−1.0 (5)	C12—C13—C14—C19	−174.2 (3)
C4—C5—C6—C10	177.9 (3)	C19—C14—C15—N2	−178.6 (3)
C5—C6—C7—C8	0.8 (5)	C13—C14—C15—N2	5.0 (5)
C10—C6—C7—C8	−178.2 (3)	C19—C14—C15—C16	2.0 (5)
C6—C7—C8—C9	0.9 (5)	C13—C14—C15—C16	−174.4 (3)
C1—N1—C9—C8	179.3 (3)	C11—N2—C15—C14	20.8 (4)
C1—N1—C9—C4	0.0 (5)	C11—N2—C15—C16	−159.8 (3)
C7—C8—C9—N1	178.4 (3)	C14—C15—C16—C17	−1.0 (5)
C7—C8—C9—C4	−2.3 (5)	N2—C15—C16—C17	179.6 (3)
C5—C4—C9—N1	−178.6 (3)	C15—C16—C17—C18	−1.0 (5)
C3—C4—C9—N1	0.7 (5)	C16—C17—C18—C19	1.9 (6)
C5—C4—C9—C8	2.0 (5)	C17—C18—C19—C14	−0.9 (5)
C3—C4—C9—C8	−178.7 (3)	C15—C14—C19—C18	−1.0 (5)
C15—N2—C11—C2	−172.8 (3)	C13—C14—C19—C18	175.3 (3)

### Hydrogen-bond geometry (Å, º)

**Table:**

*D*—H···*A*	*D*—H	H···*A*	*D*···*A*	*D*—H···*A*
C3—H3···N2	0.93	2.45	2.788 (4)	102
C11—H11···Cl1	0.98	2.72	3.074 (3)	102
C17—H17···O1^i^	0.93	2.49	3.243 (5)	138

Symmetry code: (i) *x*−1/2, *y*, −*z*+1/2.
